# Next-generation risk assessment: Integrating *in vitro* data and physiologically-based pharmacokinetic (PBPK) modeling for vancomycin nephrotoxicity evaluation

**DOI:** 10.1016/j.namjnl.2025.100018

**Published:** 2025-04-10

**Authors:** Francisco da Silva Rezende, Leonardo Pinto, Antônio Anax Oliveira Falcão, Ana Carolina Cavalheiro Paulelli, Renata Mazaro-Costa, Natália Valadares de Moraes, Fernanda de Lima Moreira

**Affiliations:** a Laboratory of Pharmacometrics (LabFarma), Faculdade de Farmácia, Universidade Federal do Rio de Janeiro, Rio de Janeiro, RJ, Brazil; b Aima Toxicologia, São Paulo, SP, Brazil; c Departamento de Farmacologia - Instituto de Ciências Biológicas - Universidade Federal de Goiás, GO, Brazil; d Center for Pharmacometrics and Systems Pharmacology, Department of Pharmaceutics, College of Pharmacy, University of Florida, Orlando, FL, USA

**Keywords:** BMDL, NAM, PDE, Toxicokinetics

## Abstract

Recognizing the need of implementing new approach methodologies (NAMs) in the pharmaceutical industry to establish health-based limits, this study employs an innovative strategy for deriving the permitted daily exposure (PDE) value, using vancomycin as a study case. We integrated *in vitro* toxicity data with physiologically based pharmacokinetic modeling-facilitated reverse dosimetry (PBPK-RD) to support setting health-based exposure limits, ensuring safety in the context of vancomycin-induced nephrotoxicity. A PBPK model for vancomycin was developed using PK-Sim v.11.3 in both rodents and humans, and its predictive performance was verified by comparing observed and simulated pharmacokinetic profiles. The PBPK-RD approach was then applied to translate *in vitro* concentration-response data into *in vivo* dose-response estimates in humans, resulting in a predicted Benchmark Dose Lower Confidence Limit (BMDL_5_) value of 0.01 mg/kg/day. This value served as the Point of Departure (PoD) for Permitted Daily Exposure (PDE) calculation, corrected for body weight of 50 kg and accounting for uncertainty factors (F). F1 and F2 were set to 1 due to using human data and incorporating interindividual variability within the model, respectively. F3 was set to 10 to address potential chronic exposure effects, while F4 and F5 were each set to 1, reflecting confidence in the dataset and the conservative nature of the PoD. This approach derived a PDE of 0.05 mg/day, lower than the traditionally calculated PDE (0.2 mg/day) based on the lowest clinical dose as the PoD. This study demonstrates the potential of PBPK-RD as a NAM-based strategy for refining toxicological assessments within the pharmaceutical industry.

## Highlights

• PBPK is a valuable tool to account for physiologic and intersubject variability.
• PBPK approach provides a reference dose of vancomycin that induces nephrotoxicity.
• PBPK modeling-facilitated reverse dosimetry provides the PoD value of 0.5 mg/day.
• Vancomycin PoD value obtained with PBPK can be applied in risk assessment.
• The PDE of 0.05 mg/day for vancomycin can be used in cleaning validation protocols.

## 1 Introduction

The clinical use of vancomycin, an antibiotic for treating Gram-positive bacterial infections, is limited by its narrow therapeutic range and nephrotoxicity (Altowayan et al., 2023; Kunming et al. 2021). Approximately 12 % of patients infected with methicillin-resistant *Staphylococcus aureus* (MRSA) treated with vancomycin experience nephrotoxicity (Yalamarti et al. 2021). Histopathology and biomarker-based assessments in animal models suggest a nephrotoxicity dependent on vancomycin dose (Murphy; Barreto 2022; Kan et al. 2022; Ngeleka et al. 1989; Papp et al. 2022). In clinical studies, nephrotoxicity has been evaluated through urinary biomarkers of vancomycin-induced acute kidney injury (Awdishu et al., 2021), clinical kidney biopsies (Ganesh et al. 2024), and altered biochemical parameters of renal function (Altowayan et al. 2023).

Besides its clinical implications, vancomycin's safety profile also raises concerns about potential cross-contamination of other drugs during manufacturing. Permitted daily exposure (PDE) is critical in cleaning validation protocols, as it establishes safe limits for residual active pharmaceutical ingredients and other potentially harmful contaminants in shared facilities. The European Medicines Agency (EMA, 2014) and the International Council for Harmonisation (ICH, 2022) have outlined guidelines for determining PDE, emphasizing the use of toxicological data and a science-based risk assessment approach to ensure thorough removal of contaminants from equipment surfaces. Setting PDE limits also aligns with Good Manufacturing Practices, helping to define the effectiveness of the cleaning process and prevent quality defects that could compromise patient safety. Traditionally a PDE is derived from NO(A)EL or LO(A)EL values obtained in sub-chronic and chronic animal studies. However, with the science-based risk assessment approach across multiple fields, non-animal testing strategies have become a priority for assessing human health risks associated with chemical exposure (Avila et al., 2020). Alternative methods, such as *in vitro* and *in silico* tools, are fundamental as they are fit-for-purpose in some decision contexts and can be faster and more cost-effective than animal models while adhering to the 3Rs principles (Replacement, Reduction, and Refinement) (Clark, 2018). Additionally, *in silico* models, such as physiologically based pharmacokinetics (PBPK), facilitate extrapolations between species, enabling simulations of pharmacokinetic profiles under various physiological conditions (Espié et al. 2009).

PBPK modeling uses a series of mathematical equations that account for physiological variables and represent a quantitative mechanistic scenario describing the absorption, distribution, metabolism, and excretion of xenobiotics, allowing the assessment of drug concentrations in the target organ (Jones and Rowland-Yeo, 2013). PBPK models translate exposure from multiple administration routes (*e.g.*, oral, dermal, pulmonary) into concentrations of the toxicant reaching target organs, such as those related to toxic effects (Sarigiannis et al. 2019). PBPK modeling-facilitated reverse dosimetry (PBPK-RD) has been used to estimate the doses of humans exposed to toxic compounds based on concentrations found in biological samples (Paini et al. 2019). This approach, termed "next-generation" PBPK within the risk assessment field (Paini et al. 2019), considers various factors, including pharmacokinetics, metabolism, and body distribution of the compound, to reconstruct the exposure scenario. Reverse dosimetry has been particularly useful in assessing exposure to environmental pollutants and occupational risks, allowing the estimation of exposure levels based on biomonitoring data (Najjar et al. 2022). Thus, the PBPK model applied to toxicological risk assessment supports the understanding and characterization of the relationship between dose metrics and biologically toxic concentrations at the target site. Our work employed an innovative approach for deriving the PDE value using vancomycin as a case study. We integrated *in vitro* toxicity data with PBPK-RD to support the establishment of safe exposure levels in humans. Although the standard approach described in the current guidelines is based on the use of points of departure from traditional animal studies, we propose an alternative way of setting a health-based limit. The benefits and limitations of this approach will be discussed, illustrating opportunities for increasing the uptake of NAMs in the pharmaceutical sector.

## 2 Methodology

### 2.1 Software

The PBPK model was developed using large molecule module of PK-Sim version 11.3 (Open Systems Pharmacology, https://www.open-systems-pharmacology.org/). Physicochemical and ADMET parameters not available in the literature were obtained from the software ADMET predictor version 11.0 (Simulation Plus, Lancaster, CA, USA). Data extraction from the literature was conducted using WebPlotDigitizer (https://automeris.io/WebPlotDigiti-zer/). Benchmark dose (BMD) modeling followed the guidelines provided by the US Environmental Protection Agency (EPA) using BMD software version 3.3.2 (https://www.epa.gov/bmds/download-bmds).

### 2.2 Vancomycin PBPK model building in humans

**Fig. 1:**
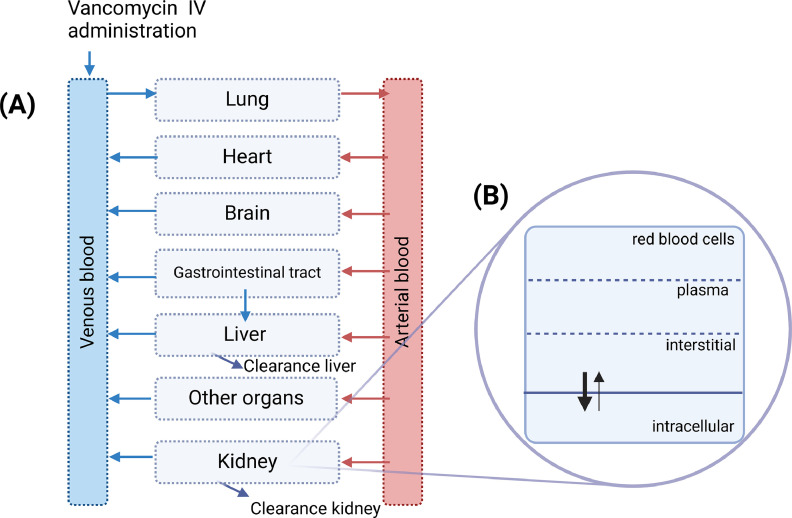
Schematic of the vancomycin full PBPK model in humans. **A)** Full physiologically based pharmacokinetic model; **B)** Kidney compartments, highlighting the increased cell permeability in the direction of intercellular to intracellular compartment.

This work followed the best practices in PBPK model development, refinement and validation reported in the current OECD guideline (OECD 2021). A full PBPK model was developed to evaluate vancomycin exposure in plasma and tissues (Fig. 1). Building on previous modeling efforts (Du et al. 2020), a PBPK model (Table 1) for vancomycin was optimized and validated using data from healthy volunteers . Virtual patients were created in PK-Sim, incorporating data on the number of individuals, female proportion, age, height, and weight according to each clinical trial (Boeckh et al., 1988, Cutler et al., 1984, Healy et al., 1987) (Table 2 and Table 3).

**Table 1:** Input parameters for human, rat and mouse physiologically based pharmacokinetic (PBPK) models for vancomycin.

	**Human**		**Rat**		**Mouse**	
**Parameter**	**Value**	**Reference**	**Value**	**Reference**	**Value**	**Reference**
Molecular weight (g/mol)	1449.265	Drugbank	1449.265	Drugbank	1449.265	Drugbank
log P	−3.10	Pubchem	−3.10	Pubchem	−3.10	Pubchem
pKa	2.18 (acid), 7.75 (base)	(Takács-Novák et al., 1993)	2.18 (acid), 7.75 (base)	(Takács-Novák et al., 1993)	2.18 (acid), 7.75 (base)	(Takács-Novák et al., 1993)
B/P	0.75	(Shin et al., 1992)	3.7	ADMET	1.05	ADMET
f_U_	0.67	(Kees et al., 2014)	0.10	ADMET	0.08	ADMET
Water solubility (mg/mL)	100	PubChem	100	Pubchem	100	Pubchem
Partition coefficient	Rodgers and Rowland	Calculated with PK-Sim	Rodgers and Rowland	Calculated with PK-Sim	Rodgers and Rowland	Calculated with PK-Sim
Cellular permeability	PK-Sim standard	Calculated with PK-Sim	PK-Sim standard	Calculated with PK-Sim	PK-Sim standard	Calculated with PK-Sim
(cm/min)^a^	0.01	Predicted with PK-Sim Parameter identification	0.01	Predicted with PK-Sim Parameter identification	0.01	Predicted with PK-Sim Parameter identification
Kidney permeability intercellular to intracellular space (cm/min)^a^	0.2	Predicted with PK-Sim Parameter identification	0.2	Predicted with PK-Sim Parameter identification	0.2	Predicted with PK-Sim Parameter identification
CL_H_	0.42 mL/min/kg	(Golper et al., 1988)	0.09 L/h/kg	(Marre et al., 1984; Ngeleka et al., 1989)	0.53 L/h/kg	(Liu et al., 2015)
CL_R_	1.17 mL/min/kg or GFR 1.0^b^	(Boeckh et al., 1988)	0.37 L/h/kg	(Marre et al., 1984; Ngeleka et al., 1989)	0.13 L/h/kg	(Liu et al., 2015)

Abbreviations: B/P: blood to plasma ratio, f_U_: plasma unbound fraction, CL_H_: Hepatic Clearance, CL_R_: Renal Clearance.

a Parameters predicted during refinement of the kidney bioaccumulation PBPK model, transforming a diffusion distribution model into a permeability model.

b The renal clearance from a clinical study (Boeckh et al. 1988), was initially used in a middle-out approach. The renal clearance set in the final model accounted for a Glomerular Filtration Rate (GFR) of 1.0.

**Table 2:** Demographic data from published clinical studies administering vancomycin to healthy volunteers used in the validation of a physiologically-based pharmacokinetic model.

Reference	n	Female proportion	Age (years)	Weight (kg)	Infusion time (min)	Posology	Systemic Clearance (L/H)	Renal clearance (L/h)	Creatinine clearance (mL/min)
(Healy et al., 1987)	11	0.36	24.7(± 2.1)	66.5 (±11.2)	60	500 mg/ 6 h or 1 g/12 h	5.0	–	110 (± 19.3)
(Boeckh et al., 1988)	10	0.50	20–50	55–78	60	500 mg or 1 g	5.7	4.6	92.7 (72.3 - 117.1)
(Cutler et al., 1984)	12	0	20–26	76.9 (72.6 – 83.2)	60	6 mg/kg	4.7	4.2	129.6

### 2.3 Establishment of a rodent physiological pharmacokinetic model and interspecies extrapolation to human renal intracellular PBPK

The PBPK model for vancomycin was built in rats and mice and verified using plasma and kidney cell exposure data (Liu et al., 2015; Marre et al., 1984; Ngeleka et al., 1989; Table S1). This additional step was necessary since renal cell exposure data in humans are lacking. The input data for interspecies vancomycin PBPK models are shown in Table 1. For the rodent PBPK models, physicochemical and ADMET parameters were the same as the human model, except for species-specific parameters: fraction unbound, blood-to-plasma ratio and hepatic and renal clearance. Hepatic and renal clearance values from experimental studies (Marre et al., 1984; Ngeleka et al., 1989; Liu et al. 2015) were used to set the rodent elimination model.

### 2.4 PBPK models refinement and validation

To refine the vancomycin PBPK model, a sensitivity analysis was conducted to evaluate which input parameters impacted the volume of distribution. The sensitivity was calculated as following:

$$S=\frac{\Delta PK}{\Delta p}\times \frac{p}{PK}$$

where *S* is the sensitivity, *PK* is the initial value of the pharmacokinetic parameter, Δ*PK* is the change of the pharmacokinetic parameter from the initial value, *p* is the initial value of the examined parameter, and Δ*p* is the change of the examined parameter from the initial value, respectively. A sensitivity of + 1.0 indicates that *a* + 10 % change of an examined input parameter causes a + 10 % change in the predicted pharmacokinetic parameter. A sensitivity analysis, including all input data, was generated and only parameters different from zero were considered relevant.

To adjust the vancomycin permeability, the parameter identification tool provided by PK-Sim®, using the Leverenberg-Marquardt algorithm was applied to find the optimal cellular permeability and kidney permeability intercellular to intracellular space values within a specific range to minimize the residuals between the simulation output and the actual observed values from the studies used during model refinement step.

Model validation was considered successful when the observed PK profile was within the 5th and 95th percentiles of predicted data, and the predicted PK parameters were within a 0.5- to 2-fold range compared to the observed data (OECD 2021).

### 2.5 Obtaining 5 % benchmark concentration lower (BMCL_5_) from *in vitro* nephrotoxicity data

The BMCL_5_ risk of nephrotoxicity was used as the *in vitro* toxicity point of departure (PoD) for *in vitro* to *in vivo* extrapolation (IVIVE), using four studies (Papp et al. 2022; Sakamoto et al. 2017; Yu et al. 2022; Yin et al., 2023). These studies evaluated renal *in vitro* cell models from three species (human, rat and porcine), focusing on cell viability after 24 or 48 h of incubation. *In vitro* experimental data were used for the BMC modeling setting the model type as continuous data. The BMCL_5_ value was determined using EPA default settings with a 95 % confidence interval (95CI) and a Benchmark Response factor (BMRF) value of 0.05, indicating a 5 % change in response relative to the background. The best dose-response model was selected based on the following criteria: if BMCL_5_ values differed by ≥ 3, the model with the lowest BMCL_5_ was chosen; if BMCL_5_ values were close, the model with the lowest Akaike information criterion (AIC) was selected. The BMCL_5_ value was employed as the maximum intracellular kidney concentration associated with a kidney toxic response in the PBPK model.

### 2.6 *In vitro* to *in vivo* extrapolation and reverse dosimetry approaches to derive *in vivo* doses related to intracellular nephrotoxicity

To convert *in vitro* PoD to *in vivo* PoD, we predicted the vancomycin *in vivo* doses, by reverse dosimetry, producing mean (5–95 confidence interval-CI) maximum kidney intracellular concentration (Cmax_intracellular,kidney_) values equivalent to observed *in vitro* (Yu et al. 2022).

The PBPK simulations employed virtual trials of 1000 individuals with 50 % female and 50 % male, ages ranging between 18 and 80 years and glomerular filtration rates ranging from 15 to 220 mL/min/1.73m^2^ receiving 14-day treatment with vancomycin via a 30-min intravenous infusion once daily. The Demographic and biochemical characteristics of the virtual individuals are depicted in Supplementary material Figure S1. The doses simulated were 0.02 mg/kg; 0.04 mg/kg; 0.08 mg/kg; 0.16 mg/kg and 0.32 mg/kg.

### 2.7 Obtaining 5 % lower benchmark dose BMDL_5_ from *in vivo* predicted doses *versus in vitro* nephrotoxicity response

The 5 % lower benchmark dose (BMDL_5_) value was derived from plotting *in vivo* predicted doses, obtained through the reverse dosimetry approach, *versus in vitro* nephrotoxicity responses from Yu et al. 2022. The BMDL_5_ value was obtained using the EPA's online tool BMDS version 3.3.2 and the criteria for model selection were the same as mentioned in the previous session. The BMDL_5_ value obtained was taken as the point of departure (PoD) in mg/kg/day vancomycin.

### 2.8 Vancomycin permitted daily exposure

The vancomycin Permitted Daily Exposure (PDE) value was calculated based on recommendations from EMA (2014) and ICH (2021;^2022^) guidelines and using the following formula:

$$PDE=\frac{PoD\times BW}{F1\times F2\times F3\times F4\times F5}$$

The PoD selected was the BMDL_5_ value in mg/kg/day obtained in the previous section, the body weight of 50 kg was used for an adult individual (EMA, 2014), F1 is the factor to account for extrapolation between species; F2 is the factor accounting for interindividual variability; F3 is the factor to account for toxicity studies of short-term exposure; F4 is the factor that may be applied in cases of severe toxicity and F5 is the factor that may be applied if the No Effect Level was not established (ICH 2021).

The PDE value in mg/daily dose obtained was considered for interindividual variability using the final PBPK model with a virtual trial of 1000 Caucasian individuals with 50 % female and 50 % male, ages ranging between 18 and 80 years and glomerular filtration rates ranging from 15 to 220 mL/min/1.73m^2^.

## 3 Results

### 3.1 Vancomycin PBPK model building in humans

**Fig. 2:**
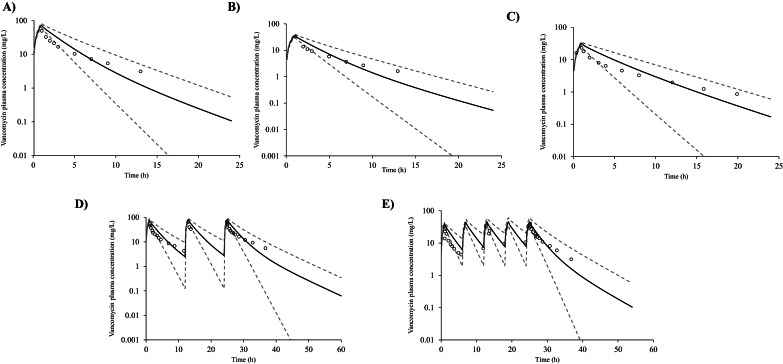
Predicted and observed plasma concentration *versus* time plot of vancomycin after a 60 min intravenous infusion in healthy volunteers. The black open circles represent the observed data from the clinical study, the solid black line represents the mean predicted values, and the dashed grey lines represent the 95 % confidence intervals. **A)**Boeck et al. 1988, 1 g vancomycin single dose; **B)**Boeck et al. 1988, 0.5 g vancomycin single dose; **C)**Cutler et al. 1984, 6 mg/kg vancomycin single dose; **D)**Healy et al. 1987, 1 g vancomycin every 12 h, 3 doses; **E)**Healy et al. 1987, 0.5 g vancomycin every 6 h, 5 doses.

The vancomycin PBPK model in healthy volunteers was developed and successfully validated by comparing observed (Cutler et al. 1984; Healy et al. 1987; Boeckh et al. 1988) and predicted plasma concentration *versus* time profiles (Fig. 2 and Table 3). The large molecule module was selected considering the high molecular weight of vancomycin. In this module, compartments of endosomes and lysosomes within vascular endothelial cells were added, besides organ-specific lymph flow was added (Niederalt et al. 2018).

**Table 3:** Evaluation of physiologically-based pharmacokinetic modeling comparing observed and predicted pharmacokinetic parameters in humans and rodents receiving vancomycin.

**Parameter**	Boeckh et al., 1988	Boeckh et al., 1988	Cutler et al., 1984	Healy et al., 1987	Healy et al., 1987	Marre et al. 1984	Ngeleka et al. 1989	Liu et al. 2015
	**Human**	**Human**	**Human**	**Human**	**Human**	**Rat**	**Rat**	**Mouse**
Dose	0.5 g	1 g	6 mg/kg	0.5 g q6h	1 g q12h	10 mg/kg	20 mg/kg	15 mg/kg
CL_obs_ (L/h)	5.7	5.8	4.7	5.1	5.2	–	–	–
CL_pred_ (L/h)	4.6	4.6	3.7	5.0	5.4	–	–	–
Ratio*	**1.2**	**1.3**	**1.3**	**1.0**	**1.0**	**–**	**–**	**–**
Cmax_plasma obs_ (mg/L)	47	74.6	23.3	40.3	65.7	–	–	–
Cmax_plasma pred_ (mg/L)	34.7	69.4	36.3	37	69.7	–	–	–
Ratio*	**1.4**	**1.1**	**0.6**	**1.1**	**0.9**	**–**	**–**	**–**
AUC_plasma obs_⁎⁎ (mgh/L)	90.7	178	98.2	116	227	36	42.8	18.9
AUC_plasma pred_⁎⁎ (mgh/L)	91.7	183.4	130.4	99.8	184.3	42.35	95.28	20.68
Ratio*	**1.0**	**1.0**	**0.8**	**1.2**	**1.2**	**0.85**	**0.45**	**0.91**
AUC_intracellular,kidney_ obs (mgh/L)	–	–	–	–	–	N.R.	217.4	80.1
AUC_intracellular,kidney_ pred (mgh/L)	–	–	–	–	–	133.07	216.4	66.91
Ratio*	**–**	**–**	**–**	**–**	**–**	**N.C.**	**1**	**1.2**

Abbreviations: AUC: Area under the curve; obs: observed; pred: predicted. N.R: Nor reported; N.C.: Not calculated.

⁎ Observed to predicted parameter value ratio.

⁎⁎ AUC zero to infinity was considered for single-dose studies, while AUC obtained in the dosing interval was considered for multiple-dose studies.

Initially, the elimination model was developed using a middle-out approach with total and renal clearances from Boeckh et al. 1988 and Golper et al. 1988. Hepatic clearance was derived from the difference between total and renal clearance. Following, the tissue and cellular distribution models available on PK-Sim were tested. Tissue-to-plasma partition coefficients were calculated using the Rodgers and Rowland equation, and cellular permeability was determined using the PK-Sim Standard equation.

Subsequently, the renal clearance was adjusted to incorporate the glomerular filtration rate fraction set as 1.0, creating a more mechanistic renal elimination model. Based on this, vancomycin renal excretion was calculated by multiplying the unbound fraction by the average glomerular filtration rate in humans. The predicted mean and upper and lower 95 % confidence interval concentration *versus* time profiles reasonably described the observed data of vancomycin after a single dose (Figs. 2A, 2B and 2C) and multiple doses (Fig. 2D and 2E) (Figure S2). The observed-to-predicted clearance (CL) ratios ranged from 1.0 to 1.3; the Cmax_plasma_ observed-to-predicted ratio ranged from 0.6 to 1.4, and AUC_plasma_ observed-to-predicted ratio ranged from 0.8 to 1.2 (Table 3), indicating that the model captures vancomycin pharmacokinetics after IV administration.

### 3.2 Establishment of a rodent physiological pharmacokinetic model and interspecies extrapolation to human renal intracellular PBPK

PBPK models were developed for rats and mice to assess vancomycin plasma and intracellular kidney exposure (Table S1). The rodent PBPK models were enhanced by incorporating an increased kidney distribution rate of vancomycin distribution from intercellular to intracellular compartments based on adjustment of the model to fit the experimental data from Ngeleka et al. 1989; Liu et al. 2015 and Boeck et al. 1988. First, the sensitivity analysis tool was applied to identify the main input data influencing on vancomycin volume of distribution (Figure S3). To adjust the vancomycin distribution to the tissues, the parameter identification tool was applied to find the optimal organ-specific permeability value within a specific range, minimizing the residuals between the simulation output and observed plasma concentrations from rodent studies. The organ-specific permeability was set to 0.01 cm/min (Figure S4). To parametrize the kidney distribution model in rodents, the kidney permeability intercellular to intracellular space value was set as 0.2 cm/min, following a parameter identification refinement step using rodent kidney intracellular vancomycin data (Figures S5 and S6, Liu et al. 2015). The vancomycin PBPK model, including the renal intracellular compartment, was back-extrapolated to humans. The Rodgers and Rowland tissue distribution equation was maintained for the three species, and the model, accounting for species-specific physiology, resulted in the mean steady-state volume of distribution of 0.22, 0.30, and 0.64 L/kg for rats, mice, and humans, respectively. The predicted volume of distribution values agreed (≤ 2-fold ratio) with the observed values of 0.37 (Ngeleka et al. 1989); 0.41 (Liu et al. 2015) and 0.62 L/kg (Boeck et al. 1988) for rat, mice and human, respectively.

The observed AUC plasma to intracellular kidney ratio (AUC_plasma_/AUC_intracellular,kidney_) in rodents showed a mean ratio of 4 to 5 (Table 3). After this optimization, the observed/predicted ratio for the AUC_intracellular,kidney_ was 1.0 for the rat model (Ngeleka et al. 1989) and 1.20 for the mouse model (Liu et al. 2015) (Table 3), demonstrating accurate predictions of intracellular kidney concentrations in rodents.

**Fig. 3:**
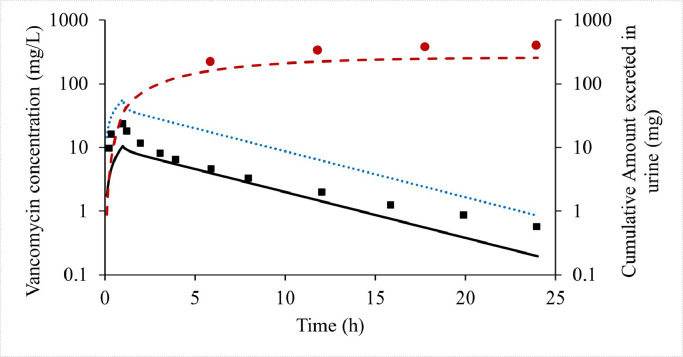
Observed and simulated vancomycin exposure in plasma, kidney intracellular, and urine, modeled using a physiologically based pharmacokinetic (PBPK) approach after a single 6 mg/kg intravenous infusion over 60 min in healthy volunteers (Cutler et al. 1984). The black solid line represents the simulated mean plasma concentration, blue dotted line represents the simulated mean intracellular kidney concentration, and red dashed line represents the simulated mean cumulative amount in urine over time. Black filled squares represent observed plasma concentration *versus* time, and red filled circles represent observed cumulative amount in urine *versus* time.

Scaling the rodent findings to humans involved extrapolating the rodent kidney distribution model while accounting for physiological interspecies differences. The strategy adopted in this study maintained the same equations for tissue and intracellular distribution used in the rodent PBPK models, but considered the species-specific physiology provided by the software for each species. Consequently, the intracellular kidney-to-plasma accumulation in rodents of ∼4-fold was extrapolated to an accumulation ratio of ∼20-fold in humans, indicating that humans are more susceptible to higher intracellular kidney vancomycin concentrations. The final PBPK model of vancomycin bioaccumulation in the human kidney was validated by comparing observed plasma and urine data from Cutler et al. (1984) (Fig. 3).

### 3.3 Obtaining 5 % benchmark concentration lower (BMCL_5_) from *in vitro* nephrotoxicity data

The BMCL_5_ value was derived from the vancomycin concentration *versus* nephrotoxicity response curves (Table 4), based on a review of the toxicological data using BMDS software (Tables S2-S9, Figures S7-S10). The study by Yu et al. 2022 (Tables S8 and S9, Figure S10) generated a BMCL_5_ value of 0.30 µM, which was further considered as the intracellular kidney concentration associated with a kidney toxic response.

**Table 4:** Five percent lower benchmark concentration (BMCL_5_) values obtained using the vancomycin dose-cell viability response curve model from *in vitro* studies.

Reference	Endpoint	Vancomycin concentration range (µM)	*In vitro* model	Specie	BMCL_5_ (µM)*
(Yu et al., 2022)	Cell viability 24 h	0.625 - 10	HK-2 cell	Human	0.30*
(Yin et al., 2023)	Cell viability 24 h	0.5 × 10^3^ - 1 × 10^3^	HK-2 cell	Human	**
(Papp et al., 2022)	Cell viability 24 h	1.9 × 10^7^ - 2.9 × 10^7^	NRK-52E cell	Rat	**
(Sakamoto et al., 2017)	Cell viability 48 h	1 × 10^3^ - 5 × 10^3^	LLC-PK1 cell	Porcine	**

Abbreviations: HK-2: human kidney immortalized proximal tubule epithelial cell line; NRK-52E: rat epithelial kidney cells; LLC-PK1: porcine proximal tubular epithelial cell line.

⁎ *p* = 0.456; **Questionable models defined by BMDS (Benchmark Dose Tools) software criteria.** Please see Supplementary material for more details. The 5 % lower benchmark dose (BMDL_5_) values shown are those obtained using the dose-response curve model that presents the lowest BMCL_5_ value, generated with the online BMDS software.

### 3.4 *In vitro* to *in vivo* extrapolation and reverse dosimetry approaches to derive *in vivo* doses related to intracellular nephrotoxicity

In the next step, a reverse dosimetry strategy was applied to generate virtual clinical trials with vancomycin administered once daily via a 30-minute intravenous infusion over fourteen days. This was done to predict the vancomycin doses in mg/kg/day to match the intracellular kidney maximum concentration related to *in vitro* kidney toxicity response (Yu et al. 2022). At the doses of 0.02; 0.04; 0.08; 0.16 and 0.32 mg/kg daily vancomycin, the mean steady state maximum intracellular kidney concentrations (Cmax_intracellular,kidney; ss_) predicted were 0.60; 1.19; 2.34; 4.68 and 8.96 µM, respectively (Table 5).

**Table 5:** *In vitro* to *in vivo* extrapolation (IVIVE) of vancomycin concentration-response to dose-response using physiologically based pharmacokinetic modeling-facilitated reverse dosimetry.

*In vivo* Dose (mg/kg) – Reverse dosimetry	*In vitro* intracellular kidney concentration (µM)	Mean Cmax_intracellular,kidney;ss_ (5–95 % confidence intervals) (µM) -In vivo intracellular kidney concentration at steady state	Response % (±standard deviation) - *In vitro* renal cell viability
0	0	–	96.85 ± 3.04
0.02	0.625	0.60 (0.47 – 0.77)	86.49 ± 4.87
0.04	1.25	1.19 (0.92 – 1.55)	80.41 ± 9.74
0.08	2.5	2.34 (1.93–2.90)	55.43 ± 4.26
0.16	5	4.68 (3.87–5.81)	26.80 ± 5.48
0.32	10	9.37 (7.74–11.61)	13.40 ± 4.87

### 3.5 Obtaining 5 % lower benchmark dose BMDL_5_ from *in vivo* predicted doses *versus in vitro* nephrotoxicity response

The BMDL_5_ was calculated by plotting the vancomycin predicted daily doses (mg/kg) against vancomycin nephrotoxicity response (Yu et al. 2022). Supplementary file demonstrated the toxicological data for the analysis using BMDS software (Tables S10 and S11, Figure S11). The BMDL_5_ value of 0.01 mg/kg (*p* = 0.524) was chosen based on the lowest AIC value provided by the Exponential M5 model. The BMDL_5_ was selected as the PoD for the critical health effect, the PoD of 0.01 mg/kg was multiplied by the body weight of 50 kg, resulting in the PoD of 0.5 mg/day.

**Fig. 4:**
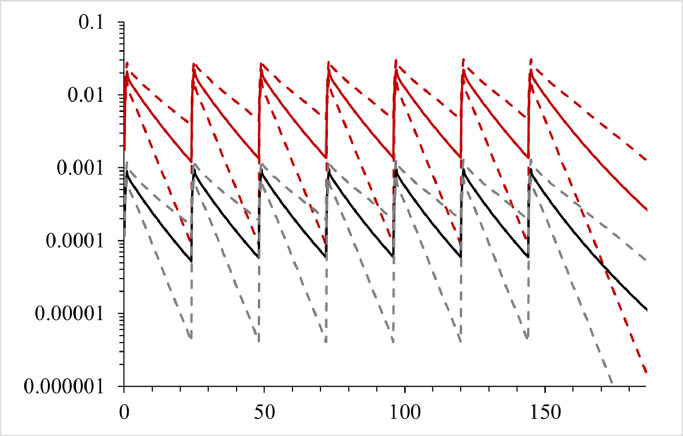
Simulated vancomycin exposure in plasma and kidney intracellular compartments after a 7-days treatment with a single daily intravenous dose of 0.05 mg, infused over 60 min, in a virtual trial of 1000 Caucasian individuals (50 % female, 50 % male), aged 18–80 years, with glomerular filtration rates ranging from 15 to 220 mL/min/1.73m^2^. The black solid line represents the simulated mean plasma concentration, the black dashed lines represent the 95 % confidence interval, the red solid line represents the simulated mean intracellular kidney concentration, and red dottd lines represent the 95 % confidence interval.

To input the interindividual variability, a clinical trial with 1000 individuals receiving an intravenous dose of 0.5 mg/day for 14 days was simulated generating mean (5–95 % confidence intervals-CI) vancomycin renal intracellular and plasma concentrations *versus* time profiles (Fig. 4). With the set of characteristics input into the model, the virtual trial accounted for interindividual variability including 1000 young and old individuals with normal to severe renal impairment function that are the main variability factors in vancomycin kidney intracellular concentrations observed with our mechanistic model.

### 3.6 Vancomycin permitted daily exposure

When applying the uncertainty factors to PDE calculation, F1 was set as 1, since the PoD was derived from human data. F2 was set as 1 since the interindividual variability was already accounted in the simulation of the exposure to vancomycin in 1000 individuals varying from 18 to 80 years old (Fig. 4), resulting in a mean (95 %CI) Cmax_intracellular,kidney_ value of 0.03 (0.02 – 0.04) µM. F3 was set as 10 to account for uncertainty due to chronic exposure to vancomycin, given that the *in vitro* study incubated renal cells only for 24 h with vancomycin and the PBPK model was also used for simulations in 7-days of drug administration. F4 was set as 1 and F5 was set as 1 due to the confidence on the available dataset and the use of a PoD which is already very conservative. The calculated PDE for vancomycin nephrotoxicity using a next-generation risk assessment approach was 0.05 mg per day.

## 4 Discussion

This study evaluated a quantitative NGRA approach for assessing vancomycin nephrotoxicity risk, utilizing PBPK modeling and simulation, interspecies extrapolation and reverse dosimetry. The PoD derived with this approach was applied in PDE calculation for future application on preventing cross-contamination in the pharmaceutical setting.

There are some examples in the literature illustrating how the combination of PBPK modeling and reverse dosimetry allow the use of *in vitro* toxicity data to support risk assessment, such as in the case of doxorubicin (Li et al. 2021), a chemotherapy agent, and troglitazone (Yu et al. 2020), a previously marketed antidiabetic drug. The PBPK model developed for doxorubicin was used to estimate its complex organism distribution and cardiotoxicity profile, which varies significantly with dosage and patient physiology. For troglitazone, PBPK modeling helped clarifying retrospectively the mechanisms behind its liver toxicity, which led to its market withdrawal. Reverse dosimetry, in both cases (Yu et al. 2020; Li et al. 2021), allowed the connection between *in vitro* toxicity data and *in vivo* exposures, supporting more accurate dose predictions, improving the safety evaluation of drugs and toxicants and supporting a move toward more human-relevant and non-animal methods in regulatory toxicology.

Evaluating vancomycin's toxicological data published, including nonclinical (O'Donnell et al. 2018; Pais et al. 2020), clinical (Kan et al. 2022; Sinha Rey et al. 2016) (Table S12) and *in vitro* Papp et al. 2022; Sakamoto et al. 2017; Yu et al. 2022; Yin et al. 2023) studies, nephrotoxicity was identified as the critical effect of vancomycin.

In the present study*, in vitro* data evaluating vancomycin nephrotoxicity in different kidney cell lines in human, porcine, and rat were assessed (Papp et al. 2022; Sakamoto et al. 2017; Yu et al. 2022; Yin et al. 2023). The results and methodologies were critically evaluated to identify fit-for-purpose *in vitro* assay data for generating *in vitro* to *in vivo* extrapolations. Three *in vitro* studies examined high vancomycin concentrations (1.9 × 10^7^ - 2.9 × 10^7^ µM - Papp et al. 2022; 1 × 10^3^ - 5 × 10^3^ µM - Sakamoto et al. 2017; 0.5 × 10^3^ - 1 × 10^3^ µM - Yin et al. 2023) showing a significant decrease in cell viability even at the lower dose tested. Evaluating cell viability as an endpoint, the BMCL_5_ values (Table 4) indicated that human kidney cell lines are more sensitive to vancomycin toxicity. Due to the fact that questionable predictive models were obtained from data modeled from studies by Yin et al., 2023 (Tables S4 and S5; Figure S8), Papp et al. 2022 (Tables S6 and S7; Figure S9) and Sakamoto et al. 2017 (Tables S8 and S9; Figure S10) and using the BMDS software, these were excluded for the next steps of vancomycin risk assessment. The study from Yu et al. 2022 evaluating vancomycin range of 0.625 – 10 µM was selected for the next step since it attended model fitting and selection according to BMDS guidance. The BMCL_5_ of 0.30 µM (Table 4; Tables S2 and S3; Figure S7) derived from human kidney immortalized proximal tubule epithelial cell (HK-2) (Yu et al. 2022) was used to guide the vancomycin toxicity in the kidney distribution PBPK model.

To bridge the relationship between vancomycin human exposure and nephrotoxicity, a PBPK model was developed and validated using plasma data from healthy volunteers (Cutler et al. 1984; Healy et al. 1987; Boeckh et al. 1988) (Fig. 2 and Table 3). The next step was to capture vancomycin concentration at the toxicity target site, the kidney cells. However, as intracellular kidney concentrations are not typically evaluated in clinical studies, only plasma and urine data, we developed a rodent kidney intracellular concentrations PBPK model and which was then extrapolated to a human PBPK model. It should be highlighted that when human biomonitoring data are scarce, or the administration route(s) and the dose administered are not well known, PBPK model refinement and validation based on animal data is a viable option (Abdullah et al. 2016; Cai et al. 2023).

The rodent PBPK model incorporated a higher-rate distribution to tissues (permeability 0.01 cm/min) and intercellular to intracellular permeability of 0.2 cm/min in kidney compartment, consistent with observed vancomycin concentrations in rodent kidney cells (Table S1; Ngeleka et al. 1989; Liu et al. 2015). While animal data supported the model development, an animal-free methodology could be developed in the future with *in vitro* input data on renal drug transporters' role in vancomycin kidney exposure. Some studies (Im et al. 2017; Li et al. 2021; Wen et al. 2018) have shown that vancomycin interacts with renal transporters and can act as an inhibitor, modulator, and substrate of drug transporters such as P-gp and OAT. However, further *in vitro* studies are needed to determine Maximum transport capacity (Jmax) and Michaelis-Menten constant (Km) values, which are essential for developing a vancomycin permeability model using PBPK and IVIVE approaches.

The next step in the PBPK model development involved extrapolating the rodent-to-human model, considering anatomical and physiological differences between species. The interspecies extrapolation incorporated species-specific parameters, such as the fraction unbound and renal and hepatic clearance (Table 1). For drug distribution extrapolation, the same tissue (Rodgers and Rowland) and cellular (PK-Sim standard) distribution models available in the software were used, accounting for interspecies differences in tissue composition, tissue volume, flow rate and others. The extrapolation was successfully validated using human plasma concentrations. Physiological differences between rodents and humans led to higher intracellular accumulation of vancomycin in human kidneys. Using the same intercellular to intracellular kidney permeability (0.2 cm/min), the predicted vancomycin intracellular concentration was approximately ∼20-fold higher than the plasma concentration (Fig. 3). A similar interspecies scaling approach using PBPK modeling and simulation was applied to predict tacrolimus nephrotoxicity (Cai et al., 2023) and vancomycin nephrotoxicity at clinical doses (Du et al., 2020). Du et al. (2020) also demonstrated a kidney total exposure about 20-fold higher than in the plasma, using a different PBPK software (Gastroplus), but similar distribution permeability and interspecies extrapolation approach.

Using the quantitative NGRA approach, we applied the vancomycin PBPK model in humans in a reverse dosimetry approach to simulate *in vivo* doses producing Cmax_intracellular,kidney_ values matching with *in vitro* kidney cell concentrations related to vancomycin nephrotoxicity. Then, the *in vivo* doses in mg/kg/day *versus in vitro* response of kidney cell viability ( %) (Yu et al. 2022) were used as input for the BMD model. The BMDL_5_ value of 0.010 mg/kg/day (*p* = 0.524) or 0.5 mg/day was stipulated as the PoD value. This PoD describes the vancomycin dose in humans generating a concentration at the target site that does not elicit a biological activity, being considered low risk for nephrotoxicity.

A PoD serves as the foundation for scientifically establishing robust Health-Based Exposure Limits (HBELs), making its selection a complex and essential process. The PoD represents a dose that elicits a specific response for the critical effect relevant to human health. Consequently, the PoD selection is important for deriving a robust PDE and supporting risk management. The PoD selected in the present study was the BMDL_5_, which is very conservative, since it accounts for the lowest benchmark dose to produce 5 % effect compared to the background. Other advantages of choosing BMDL_5_ are the measure of uncertainty, considering the lower confidence limit, and the fact that the quantitative dose-response analysis is less dependent on experimental conditions, *i.e.* the doses chosen for the study, which happens with the use of other PoDs more widely used like NO(A)EL and LO(A)ELs. Despite these advantages, the BMDL_5_ approach is less employed in the context of PDE calculation for cross-contamination (Johnson et al. 2021; Jolly et al. 2024), making it more challenging to apply the uncertainty factors for PDE calculation.

For the PDE derivation based on quantitative NGRA for vancomycin, we accounted for uncertainty factors (F). F1 and F2 were set to 1 due to the use of human data and incorporation of interindividual variability within the model, respectively. F3 was set to 10 to address potential chronic exposure effects, while F4 and F5 were each set to 1, reflecting confidence in the dataset and the conservative nature of the PoD, The PDE value obtained was 0.05 mg per day. Despite the choices made by the authors' judgment, we recognize that further work is needed to propose guidance on the application of adjustment factors when using BMDL as the PoD for non-cancer endpoints (Dourson et al. 2022).

For an exercise of the traditional PDE calculation, based on the toxicological data reviewed (Table S12), the PoD value selected was 2000 mg/day, since it is the lowest clinical dose recommended (FDA, Food and Drug Administration, 2024a) with a concentration *versus* time profile available (Boeckh et al., 1988). Setting the uncertainty factor F1 as 1, due to the dose being from the human species, and the factors F2 to F5 set as 10 each factor, the PDE calculated using traditional risk assessment approach was 0.2 mg per day.

The PDE value obtained with the NGRA approach (0.05 mg per day) is more conservative than the one obtained with the traditional approach (0.2 mg per day). The differences between the PDE values calculated using the NGRA approach and the traditional method are influenced by several factors, including data availability, PoD selection, and the assignment of variability factors (Sehner et al., 2024). Optimally, the PoD selected should be the no-effect level for the critical human health effect identified. In the traditional approach, the PoD selected was the lowest clinical dose. Nevertheless, this dose (15 mg/kg/day) causes nephrotoxicity in some individuals, particularly that with renal dysfunction (Marvin et al. 2019). Regarding the application of uncertainty factors, expert judgment plays a significant role, particularly in determining F4 and F5, as their use depends on both data availability and individual interpretation (Sehner et al., 2024). Additionally, the application of F5 varies based on whether the selected PoD is more conservative. Differences in uncertainty factor application recommendations across industry guidelines, such as ISPE (2017) and ICH Q3C (ICH, 2021) are observed.

The main limitations of this study are the scarcity of available data on vancomycin biodistribution to human kidneys and *in vitro* studies regarding the kinetics of renal drug transporters on vancomycin intracellular accumulation in humans to refine the vancomycin PBPK model. The *in vitro* study with kidney cell lines lacked information on the unbound fraction that could help to optimize the model for freely available concentration at the target site. The lack of alignment in terms of applying uncertainty factors when using BMDL as a PoD is also a limitation. Future guidelines could help to address this issue.

Currently, there are no examples in the literature on the use of *in vitro* toxicity data to support the definition of health-based exposure limits in the context of preventing cross-contamination in the pharmaceutical setting. Usually, PoDs obtained from sub-chronic and chronic toxicity studies mainly conducted in rodents are used to derive such limits. In this sense, experience about regulatory acceptance of such an approach is still lacking. Nevertheless, we propose an alternative approach to address the same problem and illustrate its application for a drug that has a well-defined critical effect. Adaptations to such approach may be required in other cases where multiple toxicity effects need to be assessed, so the most protective limits can be applied.

## 5 Conclusions

This study demonstrated that integrating *in vitro* vancomycin nephrotoxicity data and reverse dosimetry data through PBPK modeling provides a promising platform for determining the vancomycin reference dose that induces nephrotoxicity in humans. The PoD derived with the quantitative NGRA approach was applied to derive PDE value that can be applied in the future in cleaning validation protocols in order to preventing cross-contamination in the pharmaceutical setting. Compared with the traditional PDE calculation, the NGRA approach demonstrated to be more conservative. Nevertheless, in the future, regulatory agencies and pharmaceutical companies should work together to produce a standard protocol to address the uncertainty factors and the proper use of this innovative approach in the context of PDE calculation for cleaning protocols in industry.

## Glossary

BMCL5: 5 % Benchmark Concentration Lower

BMDL5: 5 % Benchmark Dose Lower

NAM: New Approach Methodology

PBPK: Physiologically based pharmacokinetics

PDE: Permitted Daily Exposure

PoD: Point of Departure

## Funding

This research did not receive any specific grant from funding agencies in the public, commercial, or not-for-profit sectors.

## Data availability statement

The datasets generated and/or analyzed during the current study are available from the corresponding author upon reasonable request.

## CRediT authorship contribution statement

**Francisco da Silva Rezende:** Writing – original draft, Validation, Methodology, Investigation, Formal analysis. **Leonardo Pinto:** Writing – review & editing, Formal analysis, Conceptualization. **Antônio Anax Oliveira Falcão:** Writing – review & editing, Formal analysis. **Ana Carolina Cavalheiro Paulelli:** Writing – review & editing, Formal analysis. **Renata Mazaro-Costa:** Writing – review & editing, Formal analysis. **Natália Valadares de Moraes:** Writing – review & editing, Formal analysis. **Fernanda de Lima Moreira:** Writing – original draft, Supervision, Software, Resources, Project administration, Investigation, Formal analysis, Data curation, Conceptualization.

## Declaration of competing interest

The authors declare that they have no known competing financial interests or personal relationships that could have appeared to influence the work reported in this paper.

## Data Availability

Data will be made available on request.

## Appendix Supplementary materials

Image, application 1
