# Supplementary material for: Next-generation risk assessment: Integrating in vitro data and physiologically-based pharmacokinetic (PBPK) modeling for vancomycin nephrotoxicity evaluation
Source: NAM J. 2025 Apr 10;1:100018. doi: 10.1016/j.namjnl.2025.100018 (PMC13289197; doi:10.1016/j.namjnl.2025.100018)
Supplement: Supplementary file 1 [file mmc1.docx]

Supplementary Material

**Next-generation risk assessment: Integrating *in vitro* data and physiologically-based pharmacokinetic (PBPK) modeling for vancomycin nephrotoxicity evaluation**

**Table S1:** Pharmacokinetic studies in rodents used to develop the vancomycin renal cell accumulation PBPK models.

| Reference | **Marre et al. 1984** | **Ngeleka et al. 1989** | **Liu et al.**  **2015** |
| --- | --- | --- | --- |
| Specie | **Rat** | **Rat** | **Mouse** |
| Dose (mg/kg) | 10 | 20 | 15 |
| AUC_plasma_ (mg.h/L) | 36 | 42.8 | 18.9 |
| AUC_intracellular,kidney_ (mg.h/L) | N.R. | 217.4 | 80.1 |
| AUC ratio | N.R. | 5.1 | 4.2 |

Abbreviation: AUC: Area under the curve, N.R.: Not reported.


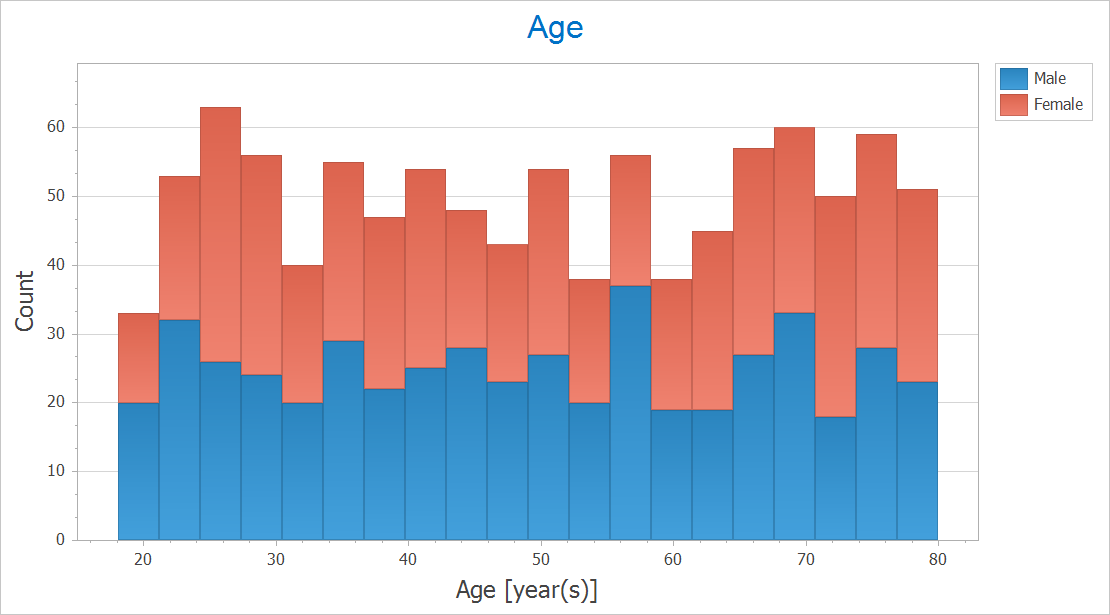

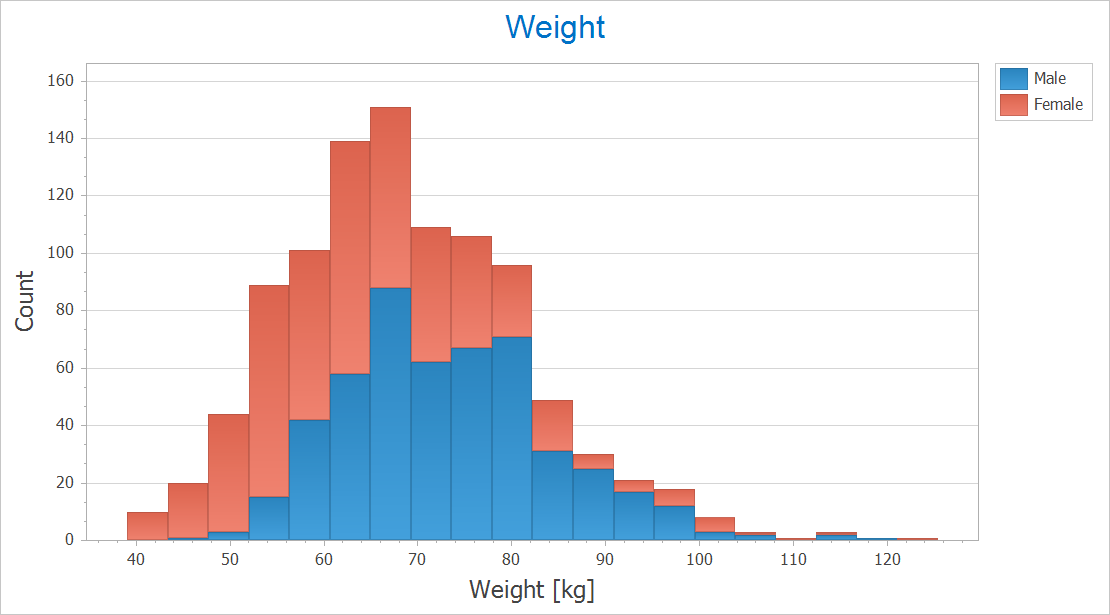


**B)**

**A)**


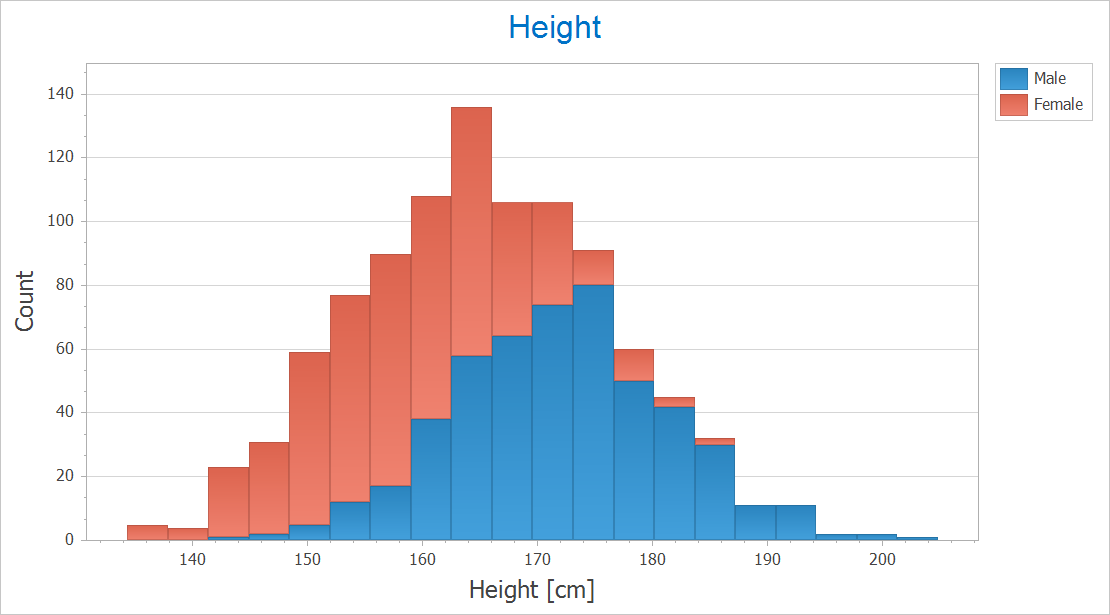


**C)**


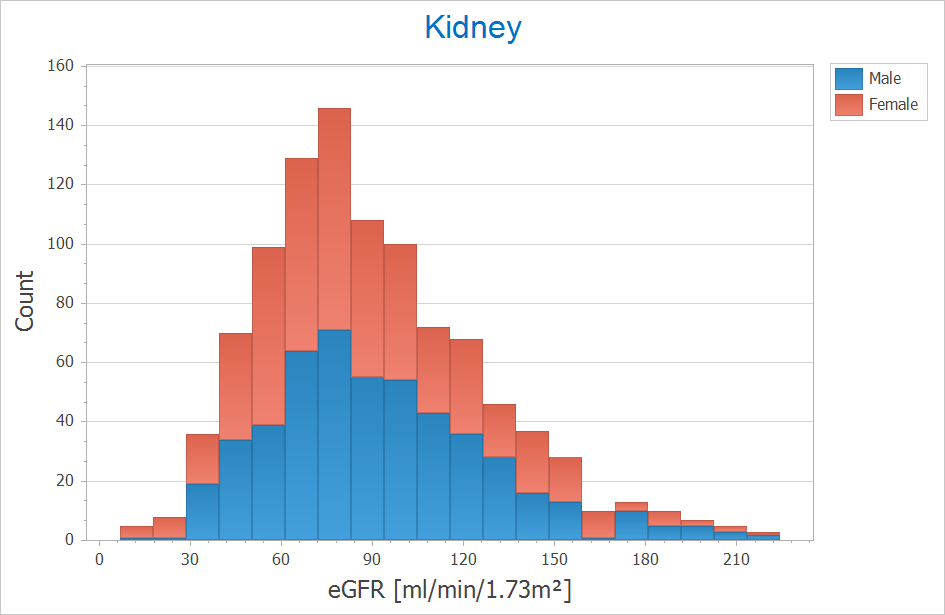


**D)**


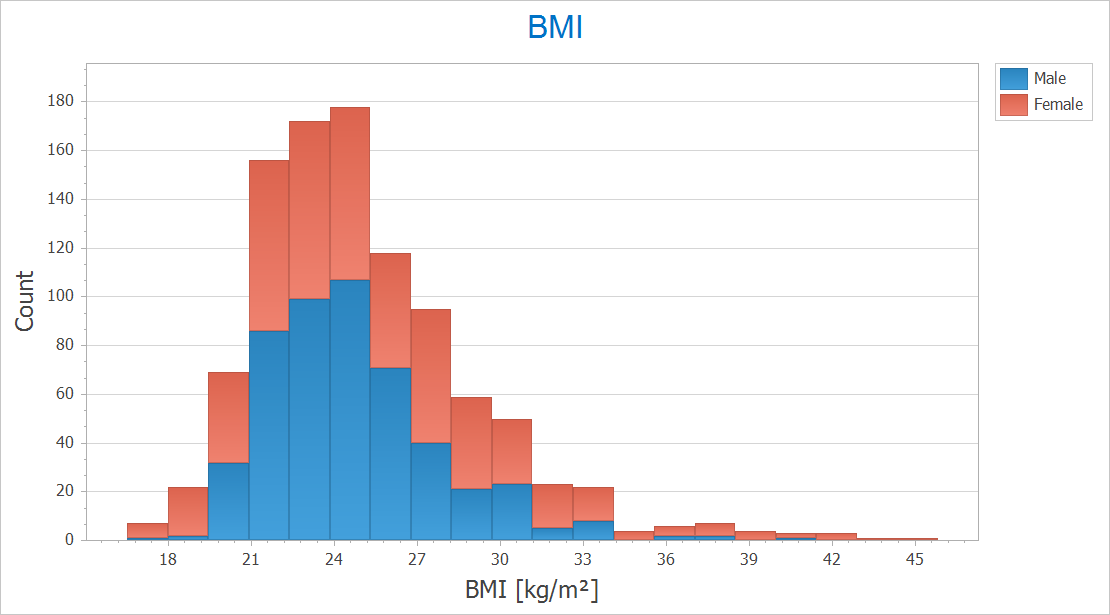


**E)**

**Figure S1:** Demographic and biochemical parameters distribution in the virtual population of 1000 individuals simulated with the vancomycin PBPK model in humans. A) Age (years); B) body weight (kg); C) height (cm); D) estimated glomerular filtration rate (eGFR) (ml/min/1.73m^2^); E) Body mass index (BMI) (kg/m^2^).


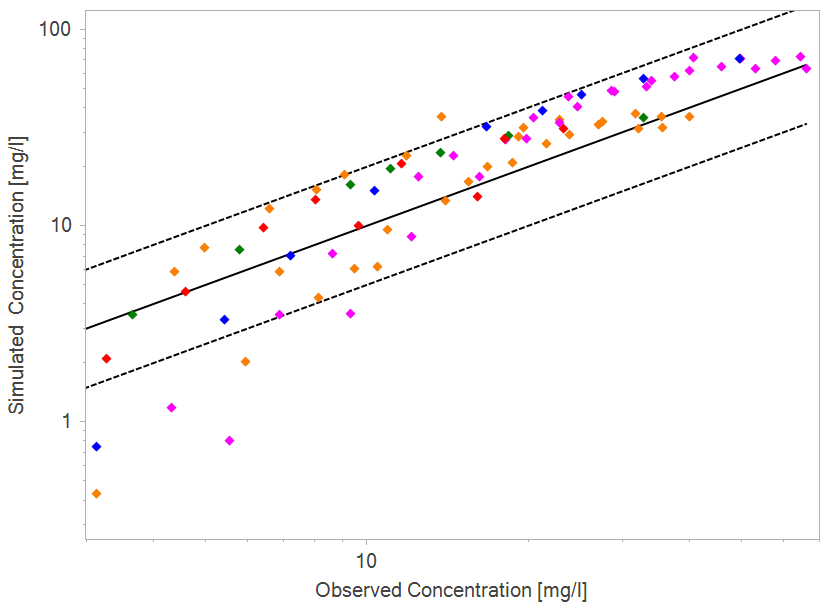


**Figure S2:** Model performance evaluation of vancomycin PBPK model in humans. The plot of the model generated versus observed concentration along with the identity line. The solid line indicates the line of unity, the dashed lines indicate a 2-fold range. The diamonds represent observed vancomycin plasma concentration and each colour represents a different study: Green diamonds: Boeckh et al. 1988 0.5 g; Blue diamonds: Boeckh et al. 1988 1g; Red diamonds: Cutler et al. 1984 6 mg/kg; orange diamonds: Healy et al. 1987 0.5g q6h; Pink diamonds: Healy et al. 1988 1g q12h.


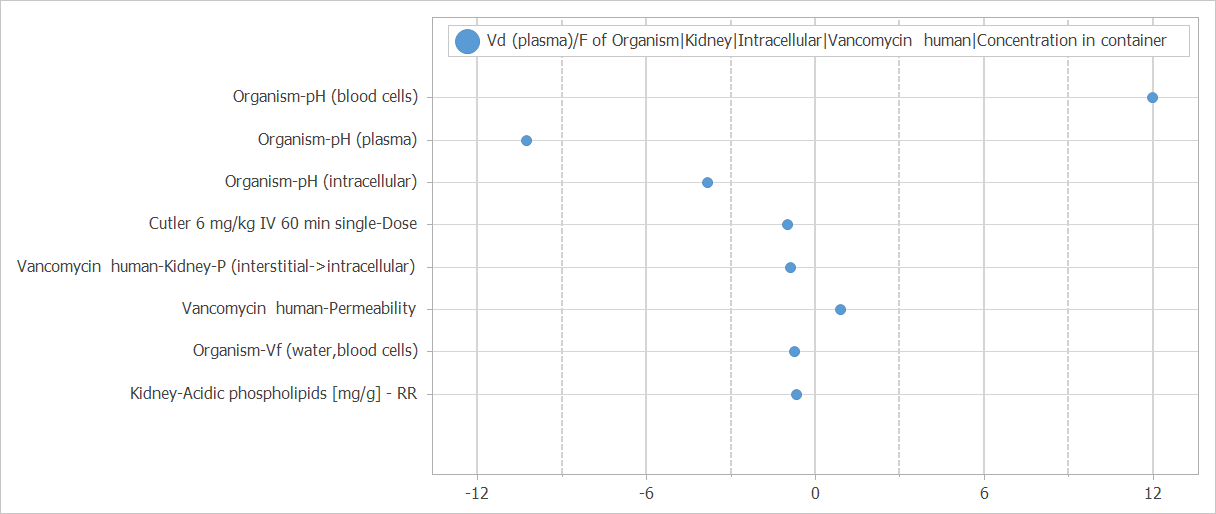


**Figure S3:** Sensitivity analysis for input parameters influencing vancomycin volume of distribution. A sensitivity of 0 indicates that the respective input parameter does not alter the pharmacokinetic parameter, a sensitivity of + 1.0 indicates that a + 10% change of an examined input parameter causes a + 10% change in the predicted pharmacokinetic parameter value, and a sensitivity of -1.0 implies that a 10% increase of the parameters leads to a 10% decrease of the pharmacokinetic parameter value.


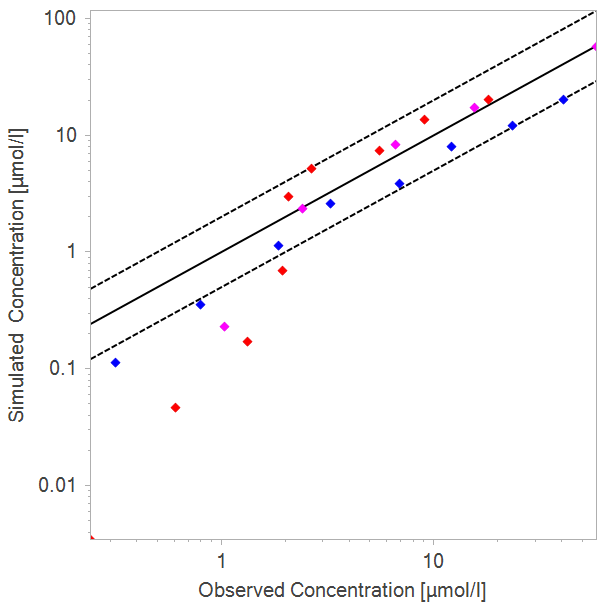


**Figure S4:** Model performance evaluation after refinement using parameter estimation tool provided by PK-Sim®. The refinement was based on the rodent plasma observed data. The organ-tissue permeability was set to 0.01 cm/min. The plot of the model generated versus observed concentration along with the identity line. The solid line indicates the line of unity, the dashed lines indicate a 2-fold range. The diamonds represent observed vancomycin plasma concentration and each colour represents a different study: Red diamonds: Liu et al. 2015; Blue diamonds: Marre et al. 1984; Pink diamonds: Ngeleka et al. 1989.


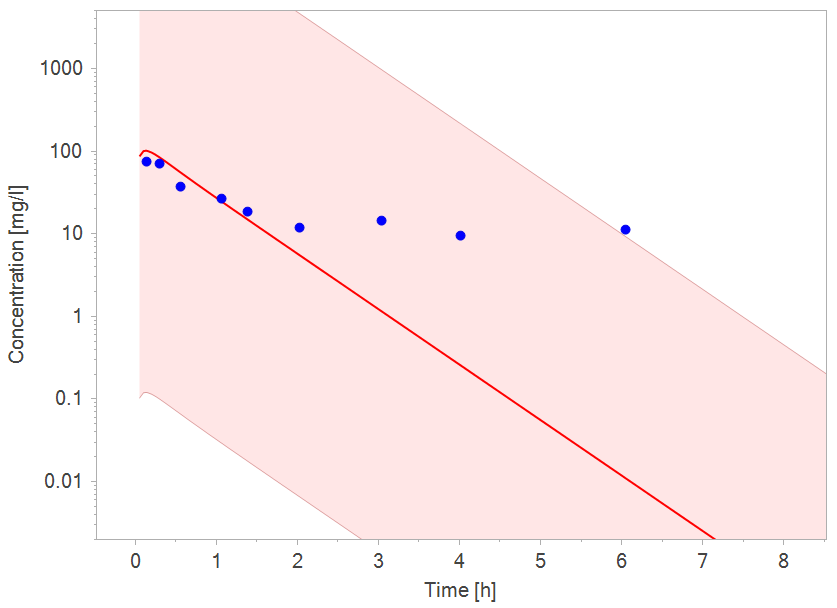


**Figure S5:** Model performance evaluation after refinement using parameter estimation tool provided by PK-Sim®. The refinement was based on the rodent renal intracellular observed data. The permeability interstitial to intracellular flux was set to 0.2 cm/min. Visual predictive check of vancomycin intracellular renal concentration versus time plot. The 5% and 95% percentiles (red light lines) and the median (red line) of the simulation are compared against the observed (blue circles) data from Liu et al. 2015.


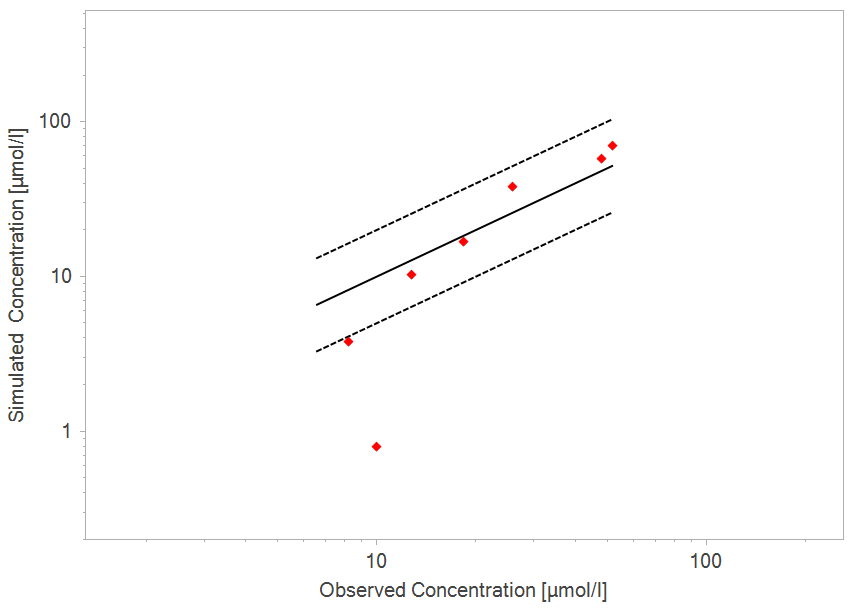


**Figure S6:** Model performance evaluation after refinement using parameter estimation tool provided by PK-Sim®. The refinement was based on the the rodent renal intracellular observed data. The permeability interstitial to intracellular flux was set to 0.2 cm/min. The plot of the model generated versus observed concentration along with the identity line. The solid line indicates the line of unity, the dashed lines indicate a 2-fold range. The red diamonds represent observed vancomycin intracellular kidney concentration from Liu et al. 2015.

The following results were generated using the EPA’s online tool BMDS version 3.3.2.

**Table S2**: Input dataset for vancomycin benchmark concentration calculation from in vitro study of Yu et al. 2022 of vancomycin concentration versus percentage of renal cells viability.

| **Concentration (µM)** | **N** | **Mean (%)** | **Std. Dev. (%)** |
| --- | --- | --- | --- |
| 0.0 | 3 | 96.95 | 3.04 |
| 0.625 | 3 | 86.49 | 4.87 |
| 1.25 | 3 | 80.4 | 9.74 |
| 2.5 | 3 | 55.4 | 4.26 |
| 5.0 | 3 | 26.8 | 5.48 |
| 10.0 | 3 | 13.4 | 4.87 |

**Table S3:** Model summary for vancomycin benchmark concentration calculation from in vitro study of Yuet al. 2022 of vancomycin concentration versus percentage of renal cells viability.

| Model | BMDL | BMD | BMDU | *P*-Value | AIC | Scaled residual at Control | Scaled residual near BMD | Recommendation and notes |
| --- | --- | --- | --- | --- | --- | --- | --- | --- |
| Exponential 3 | 0.202 | 0.287 | 0.457 | 0.067 | 122.043 | -0.369 | -0.369 | **Questionable** lowest dose/BMDL ratio > 3.0 Goodness of fit p-value < 0.1 Control stdev. fit > 1.5 |
| Exponential 5^a^ | 0.294 | 0.474 | 0.705 | 0.456 | 118.449 | 0.319 | -0.88 | **Recommended - Lowest AIC** Control stdev. fit > 1.5 |
| Hill | 0.451 | 0.596 | 0.814 | 0.285 | 119.385 | 0.641 | -1.173 | **Viable** Control stdev. fit > 1.5 |
| Power | 0.458 | 0.514 | 0.674 | <0.001 | 145.606 | 1.426 | 0.662 | **Questionable** Goodness of fit p-value < 0.1 Control stdev. fit > 1.5 |
| Linear | 0.458 | 0.514 | 0.589 | <0.001 | 145.606 | 1.426 | 0.662 | **Questionable** Goodness of fit p-value < 0.1 Control stdev. fit > 1.5 |

^a^BMDS recommended best-fitting model

Abbreviations: BMDS: Benchmark dose software; BMDL: lower benchmark dose; BMD: benchmark dose; BMDU: benchmark dose upper; AIC: Akaike information criterion.


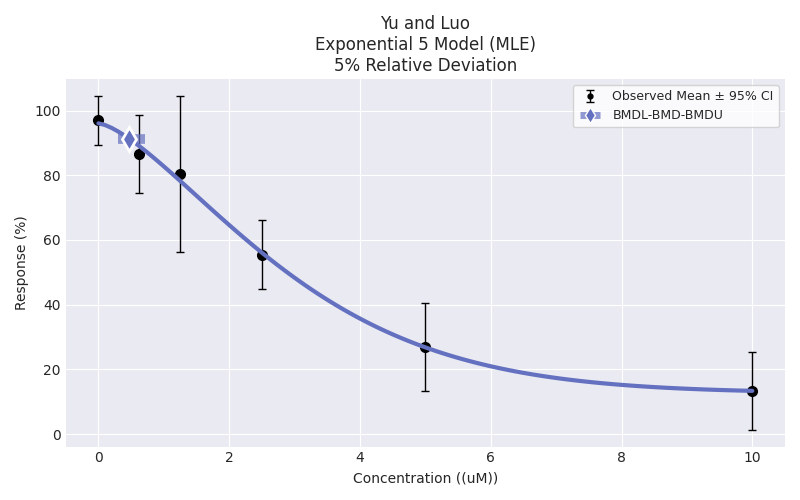


**Figure S7:** Exponential 5 Model selected to estimate 5% lower benchmark concentration for vancomycin from study of Yu et al. 2022.

**Table S4**: Input dataset for vancomycin benchmark concentration calculation from in vitro study of Yin et al. 2023 of vancomycin concentration versus percentage of renal cells viability.

| **Concentration (µM)** | **N** | **Mean (%)** | **Std. Dev. (%)** |
| --- | --- | --- | --- |
| 0 | 3 | 99.57 | 0 |
| 500 | 3 | 86.07 | 0 |
| 1000 | 3 | 85.94 | 0 |
| 2000 | 3 | 84.78 | 0 |

**Table S5:** Models Summary for vancomycin benchmark concentration calculation from in vitro study of Yin et al. 2023 of vancomycin concentration versus percentage of renal cells viability.

| Model | BMDL | BMD | BMDU | *P*-Value | AIC | Scaled Residual at Control | Scaled Residual near BMD | Recommendation and Notes |
| --- | --- | --- | --- | --- | --- | --- | --- | --- |
| Exponential 3 | 495.779 | 719.281 | 1293.97 | 0 | 73.342 | 2.111 | -2.295 | **Questionable** \|Residual near BMD\| > 2.0 Residual at control > 2.0 Goodness of fit p-value < 0.1 Control stdev. fit > 1.5 Constant variance test failed (Test 2 p-value < 0.05) |
| Exponential 5 | 56.341 | 75.662 | 207.703 | 0 | 19.764 | 0.009 | 0.009 | **Questionable** lowest dose/BMDL ratio > 3.0 lowest dose/BMD ratio > 3.0 Goodness of fit p-value < 0.1 Control stdev. fit > 1.5 Constant variance test failed (Test 2 p-value < 0.05) |
| Hill | 22.834 | 30.758 | 45.415 | 0 | 11.856 | 0.01 | 0.01 | **Questionable** lowest dose/BMDL ratio > 3.0 lowest dose/BMDL ratio > 10.0 lowest dose/BMD ratio > 3.0 lowest dose/BMD ratio > 10.0 Goodness of fit p-value < 0.1 Control stdev. fit > 1.5 Constant variance test failed (Test 2 p-value < 0.05) |
| Polynomial 2 | 546.628 | 779.423 | 1397.629 | 0 | 73.845 | 2.196 | -1.014 | **Questionable** Residual at control > 2.0 Goodness of fit p-value < 0.1 Control stdev. fit > 1.5 Constant variance test failed (Test 2 p-value < 0.05) |
| Polynomial 3 | 546.39 | 771.891 | 1397.839 | 0 | 73.845 | 2.172 | -1.011 | **Questionable** Residual at control > 2.0 Goodness of fit p-value < 0.1 Control stdev. fit > 1.5 Constant variance test failed (Test 2 p-value < 0.05) |
| Power | 546.595 | 780.66 | 1397.705 | 0 | 73.845 | 2.209 | -1.016 | **Questionable** Residual at control > 2.0 Goodness of fit p-value < 0.1 Control stdev. fit > 1.5 Constant variance test failed (Test 2 p-value < 0.05) |
| Linear | 546.646 | 777.501 | 1397.625 | 0 | 73.845 | 2.19 | -1.013 | **Questionable** Residual at control > 2.0 Goodness of fit p-value < 0.1 Control stdev. fit > 1.5 Constant variance test failed (Test 2 p-value < 0.05) |

Abbreviations: BMDS: Benchmark dose software; BMDL: lower benchmark dose; BMD: benchmark dose; BMDU: benchmark dose upper; AIC: Akaike information criterion.


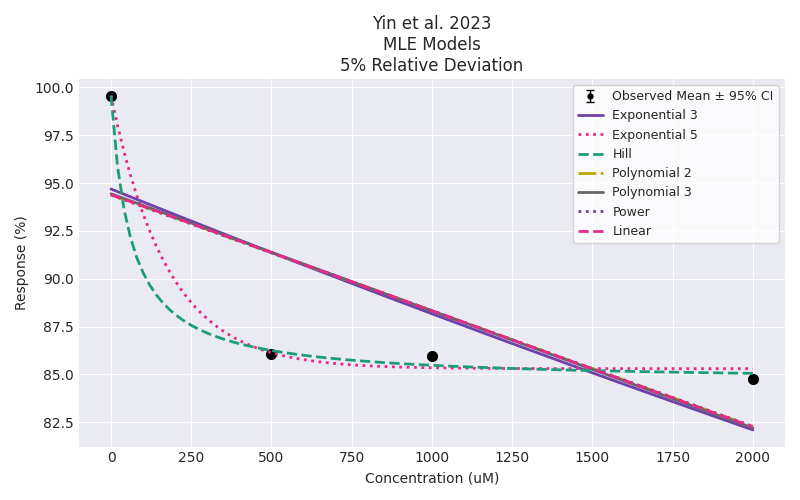


**Figure S8:** Models evaluated to estimate 5% lower benchmark concentration for vancomycin from study of Yin et al. 2023.

**Table S6**: Input dataset for vancomycin benchmark concentration calculation from in vitro study of Papp et al. 2022 of vancomycin concentration versus percentage of renal cells viability.

| **Concentration (µM)** | **N** | **Mean (%)** | **Std. Dev. (%)** |
| --- | --- | --- | --- |
| 0 | 6 | 99.52 | 7.11 |
| 1862839.8 | 6 | 24.88 | 12.79 |
| 2345798.3 | 6 | 36.96 | 39.81 |
| 2966744.9 | 6 | 19.19 | 19.19 |

**Table S7:** Models Summary for vancomycin benchmark concentration calculation from in vitro study of Papp et al. 2022 of vancomycin concentration versus percentage of renal cells viability.

| Model | BMDL | BMD | BMDU | *P*-Value | AIC | Scaled Residual at Control | Scaled Residual near BMD | Recommendation and Notes |
| --- | --- | --- | --- | --- | --- | --- | --- | --- |
| Exponential 3 | 68329 | 92039 | 428998 | 0.625 | 221.42 | 0.082 | 0.082 | **Questionable** lowest dose/BMDL ratio > 3.0 lowest dose/BMDL ratio > 10.0 lowest dose/BMD ratio > 3.0 lowest dose/BMD ratio > 10.0 Control stdev. fit > 1.5 Constant variance test failed (Test 2 p-value < 0.05) |
| Exponential 5 | 19145.505 | 279654 | 1.6E+06 | - | 226.906 | <0.001 | <0.001 | **Questionable** lowest dose/BMDL ratio > 3.0 lowest dose/BMDL ratio > 10.0 lowest dose/BMD ratio > 3.0 Zero degrees of freedom; saturated model Control stdev. fit > 1.5 Constant variance test failed (Test 2 p-value < 0.05) BMD/BMDL ratio > 3.0 |
| Hill | 69.822 | 27028.909 | 1.1E+06 | 0.163 | 224.754 | 0.002 | 0.002 | **Questionable** lowest dose/BMDL ratio > 3.0 lowest dose/BMDL ratio > 10.0 lowest dose/BMD ratio > 3.0 lowest dose/BMD ratio > 10.0 Control stdev. fit > 1.5 Constant variance test failed (Test 2 p-value < 0.05) BMD/BMDL ratio > 3.0 BMD/BMDL ratio > 20.0 |
| Polynomial 2 | 207842 | 212626 | 216979 | 0.01 | 230.086 | 1.963 | 1.963 | **Questionable** lowest dose/BMDL ratio > 3.0 lowest dose/BMD ratio > 3.0 Goodness of fit p-value < 0.1 Control stdev. fit > 1.5 Constant variance test failed (Test 2 p-value < 0.05) |
| Polynomial 3 | 149921 | 172387 | 278010 | 0.069 | 226.164 | 0.559 | 0.559 | **Questionable** lowest dose/BMDL ratio > 3.0 lowest dose/BMDL ratio > 10.0 lowest dose/BMD ratio > 3.0 lowest dose/BMD ratio > 10.0 Goodness of fit p-value < 0.1 Control stdev. fit > 1.5 Constant variance test failed (Test 2 p-value < 0.05) |
| Power | 149923 | 172332 | 367130 | 0.069 | 226.164 | 0.556 | 0.556 | **Questionable** lowest dose/BMDL ratio > 3.0 lowest dose/BMDL ratio > 10.0 lowest dose/BMD ratio > 3.0 lowest dose/BMD ratio > 10.0 Goodness of fit p-value < 0.1 Control stdev. fit > 1.5 Constant variance test failed (Test 2 p-value < 0.05) |
| Linear | 149923 | 172332 | 207883 | 0.069 | 226.164 | 0.556 | 0.556 | **Questionable** lowest dose/BMDL ratio > 3.0 lowest dose/BMDL ratio > 10.0 lowest dose/BMD ratio > 3.0 lowest dose/BMD ratio > 10.0 Goodness of fit p-value < 0.1 Control stdev. fit > 1.5 Constant variance test failed (Test 2 p-value < 0.05) |

Abbreviations: BMDS: Benchmark dose software; BMDL: lower benchmark dose; BMD: benchmark dose; BMDU: benchmark dose upper; AIC: Akaike information criterion.


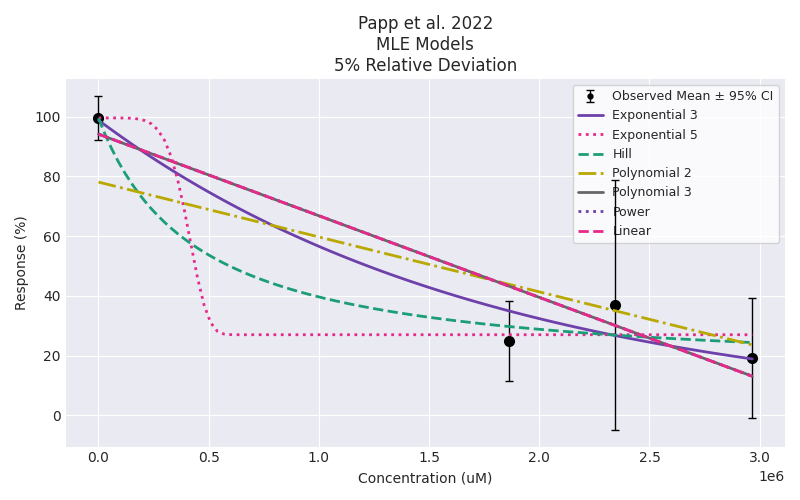


**Figure S9:** Models evaluated to estimate 5% lower benchmark concentration for vancomycin from study of Papp et al. 2022.

**Table S8**: Input dataset for vancomycin benchmark concentration calculation from in vitro study of Sakamoto et al. 2017 of vancomycin concentration versus percentage of renal cells viability.

| **Concentration (µM)** | **N** | **Mean (%)** | **Std. Dev. (%)** |
| --- | --- | --- | --- |
| 0 | 4 | 99.57 | 0 |
| 1000 | 4 | 97.41 | 0 |
| 2000 | 4 | 90.08 | 0 |
| 3000 | 4 | 65.51 | 0 |
| 4000 | 4 | 29.31 | 0 |
| 5000 | 4 | 1.72 | 0 |

**Table S9:** Models Summary for vancomycin benchmark concentration calculation from in vitro study of Sakamoto et al. 2017 of vancomycin concentration versus percentage of renal cells viability.

| Model | BMDL | BMD | BMDU | *P*-Value | AIC | Scaled Residual at Control | Scaled Residual near BMD | Recommendation and Notes |
| --- | --- | --- | --- | --- | --- | --- | --- | --- |
| Exponential 3 | 1742.334 | 1800.746 | 1859.622 | 0 | 93.73 | 1.715 | -1.186 | **Questionable** Goodness of fit p-value < 0.1 Control stdev. fit > 1.5 |
| Exponential 5 | 1742.335 | 1800.746 | 1859.619 | 0 | 95.73 | 1.715 | -1.186 | **Questionable** Goodness of fit p-value < 0.1 Control stdev. fit > 1.5 |
| Hill | 1727.939 | 1744.719 | 1761.331 | 0 | 47.71 | 3.247 | -1.236 | **Questionable** Residual at control > 2.0 Goodness of fit p-value < 0.1 Control stdev. fit > 1.5 |
| Power | 975.934 | 1098.417 | 1226.755 | 0 | 138.234 | -1.261 | -0.127 | **Questionable** Goodness of fit p-value < 0.1 Control stdev. fit > 1.5 |
| Linear | 261.967 | 280.8 | 303.139 | 0 | 190.898 | -2.748 | -2.748 | **Questionable** \|Residual near BMD\| > 2.0 lowest dose/BMDL ratio > 3.0 lowest dose/BMD ratio > 3.0 Residual at control > 2.0 Goodness of fit p-value < 0.1 Control stdev. fit > 1.5 |

Abbreviations: BMDS: Benchmark dose software; BMDL: lower benchmark dose; BMD: benchmark dose; BMDU: benchmark dose upper; AIC: Akaike information criterion.


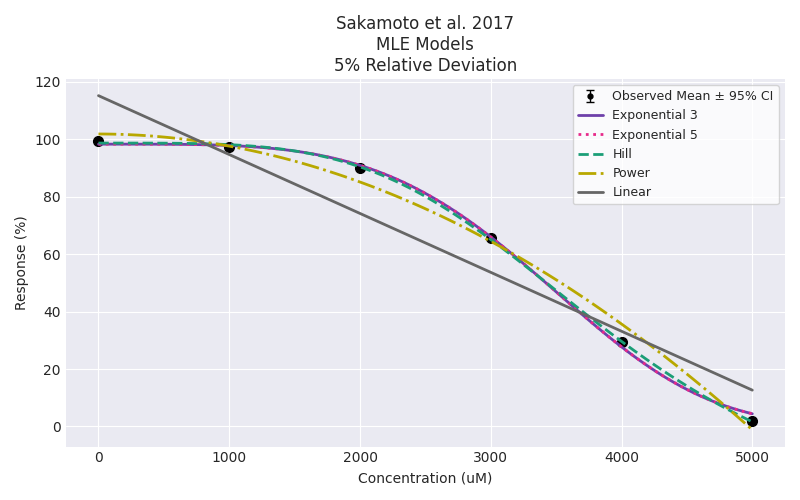


**Figure S10:** Models evaluated to estimate 5% lower benchmark concentration for vancomycin from study of Sakamoto et al. 2017.

*Obtaining 5% lower benchmark dose BMDL5 from in vivo predicted doses versus in vitro nephrotoxicity response*

**Table S10**: Input dataset for vancomycin benchmark dose calculation from in vivo predicted doses versus in vitro nephrotoxicity response.

| Dose (mg/kg) | N | Mean (%) | Std. Dev. (%) |
| --- | --- | --- | --- |
| 0 | 3 | 96.85 | 3.04 |
| 0.02 | 3 | 86.94 | 4.87 |
| 0.04 | 3 | 80.41 | 9.74 |
| 0.08 | 3 | 55.43 | 4.26 |
| 0.16 | 3 | 26.8 | 5.48 |
| 0.32 | 3 | 13.4 | 4.87 |

**Table S11:** Models Summary for vancomycin benchmark dose calculation from in vivo predicted doses (reverse dosimetry approach) versus in vitro nephrotoxicity response.

| Model | BMDL | BMD | BMDU | *P*-Value | AIC | Scaled Residual at Control | Scaled Residual near BMD | Recommendation and Notes |
| --- | --- | --- | --- | --- | --- | --- | --- | --- |
| Exponential 3 | 0.006 | 0.009 | 0.015 | 0.067 | 122.038 | -0.412 | -0.412 | **Questionable** lowest dose/BMDL ratio > 3.0 Goodness of fit p-value < 0.1 Control stdev. fit > 1.5 |
| Exponential 5^a^ | 0.01 | 0.015 | 0.023 | 0.524 | 118.17 | 0.269 | -0.777 | **Recommended - Lowest AIC** Control stdev. fit > 1.5 |
| Hill | 0.015 | 0.019 | 0.027 | 0.347 | 118.996 | 0.601 | -1.084 | **Viable** Control stdev. fit > 1.5 |
| Polynomial 2 | 0.015 | 0.017 | 0.02 | <0.001 | 145.852 | 1.438 | 0.772 | **Questionable** Goodness of fit p-value < 0.1 Control stdev. fit > 1.5 |
| Polynomial 3 | - | 0.046 | - | <0.001 | 165.521 | 1.786 | 0.577 | **Unusable** Did not successfully execute. |
| Power | 0.015 | 0.016 | 0.022 | <0.001 | 145.666 | 1.357 | 0.688 | **Questionable** Goodness of fit p-value < 0.1 Control stdev. fit > 1.5 |
| Linear | 0.015 | 0.016 | 0.019 | <0.001 | 145.658 | 1.394 | 0.714 | **Questionable** Goodness of fit p-value < 0.1 Control stdev. fit > 1.5 |

Abbreviations: BMDS: Benchmark dose software; BMDL: lower benchmark dose; BMD: benchmark dose; BMDU: benchmark dose upper; AIC: Akaike information criterion.


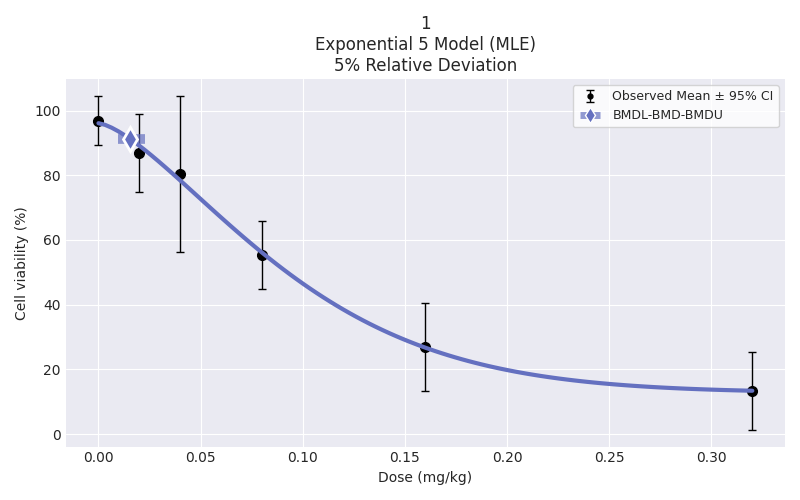


**Figure S11:** Exponential 5 Model selected for the estimation of 5% lower benchmark dose for vancomycin.

**Table S12:** Summary of the selected toxicity studies for vancomycin risk assessment.

| **Type of Study (critical effect)** | **Animal (specie)/Human** | **Number of animals/participants** | **Administration route/ doses or concentrations** | **Duration of the study** | **Potential Point of Departure – Dose-Type** | **Reference** |
| --- | --- | --- | --- | --- | --- | --- |
| LD50 | Rat | 50 | IV/ N.I. | Acute | 319 mg/kg- LD50 | Wold, Turnipseed, 1981 |
| LD50 | Rat | 50 | IP/ N.I. | Acute | 2,218 mg/kg - LD50 | Wold, Turnipseed, 1981 |
| LD50 | Mouse | 50 | IV/ N.I. | Acute | 489 mg/kg- LD50 | Wold, Turnipseed, 1981 |
| LD50 | Mouse | 50 | IP/ N.I. | Acute | 1,734 mg/kg- LD50 | Wold, Turnipseed, 1981 |
| LD50 | Mouse | 50 | SC/ N.I. | Acute | 5000 mg/kg (considering as the highest dose administered without toxicity)- LD50 | Wold, Turnipseed, 1981 |
| LD50 | Mouse | 50 | Oral/ N.I. | Acute | 5000 mg/kg (considering as the highest dose administered without toxicity)- LD50 | Wold, Turnipseed, 1981 |
| Subchronic toxicity (weight loss) | Rat | N.I. | SC/ 100, 200, 400 mg/kg daily doses | Subchronic 7 months | 400 mg/kg (considering as the highest dose administered without toxicity) - NOAEL | Wold, Turnipseed, 1981 |
| Developmental toxicity | Rat | 25 each group at 6-15 gestation days | IV/ 40,120, 200 mg/kg | 20^th^ gestational day | 200 mg/kg – developmental NOAEL | Byrd, Gries, Buening 1994 |
| Developmental toxicity | Rabbit | 20 each group at 6-18 gestation days | IV/ 40, 80, 120 mg/kg | 28^th^ gestational day | 80 mg/kg – developmental NOAEL | Byrd, Gries, Buening 1994 |
| TD50 (nephrotoxicity) | Rat | Varied (4-12 each group) | IP/ 50 – 300 mg/kg | Acute (1-14 days) | 130 mg/kg – TD50 | O’Donnel et al. 2018 |
| Minimum therapeutic dose in humans | Human | N.A. | IV/15 mg/kg | N.A. | 15 mg/kg - LOAEL | FDA Label |
| High doses (nephrotoxicity) | Human | 26 for group ≥4 g/day and 220 for group < 4 g/day | IV/ < 4 g/day, ≥ 4 g/day | Acute | 4g/day (body weight 70 kg): 57 mg/kg – LOAEL? | Lodise et al. 2008 |
| BMDL_5_ (nephrotoxicity) | Virtual Human | 100 | IV/ 0.02; 0.04; 0.08  0.16; 0.32 mg/kg | Acute (14 days) | 0.012 mg/kg – BMDL_5_ | Present study |

Abbreviations: LD50: 50% of letal dose; TD50: 50% of toxic dose, BMDL_5_: 5% lower benchmark dose; LOAEL: Lowest Observed Adverse Effect Level; NOAEL: No Observed Adverse Effect Level; IV: intravenous, IP: intraperitoneal; SC: subcutaneous; NI: not informed, N.A.: not applied.
